# Recent advancements in nanomaterial-laden contact lenses for diagnosis and treatment of glaucoma, review and update

**DOI:** 10.1186/s12951-023-02166-w

**Published:** 2023-11-02

**Authors:** Roghayyeh Baghban, Mohammad Reza Talebnejad, Aidin Meshksar, Mojtaba Heydari, Mohammad Reza Khalili

**Affiliations:** Poostchi Ophthalmology Research Center, Department of Ophthalmology, School of Medicine, Shiraz University of Medical Sciences, Shiraz, Iran

**Keywords:** Glaucoma, Contact lenses, Drug delivery system, Diagnosis, Treatment

## Abstract

Despite the existence of numerous eye drops in the market, most of them are not sufficiently effective because of quick clearance and the barriers within the eye. To increase the delivery of the drugs to the eye, various new formulations have been explored in recent decades. These formulations aim to enhance drug retention and penetration, while enabling sustained drug release over extended periods. One such innovative approach is the utilization of contact lenses, which were originally designed for cosmetic purposes and vision correction. Contact lenses have appeared as a promising formulation for ocular drug delivery, as they can increase the bioavailability of drugs in the eye and diminish unwanted side effects. They are specifically appropriate for treating chronic eye conditions, making them an area of interest for researchers in the field of ophthalmology. This review outlines the promising potential of nanomaterial-laden contact lenses for diagnosis and treatment of glaucoma. It classifies therapeutic approaches based on nanomaterial type, summarizes diagnostic advances, discusses improvement of contact lenses properties, covers marketing perspectives, and acknowledges the challenges of these innovative contact lenses for glaucoma management.

## Introduction

**Fig. 1:**
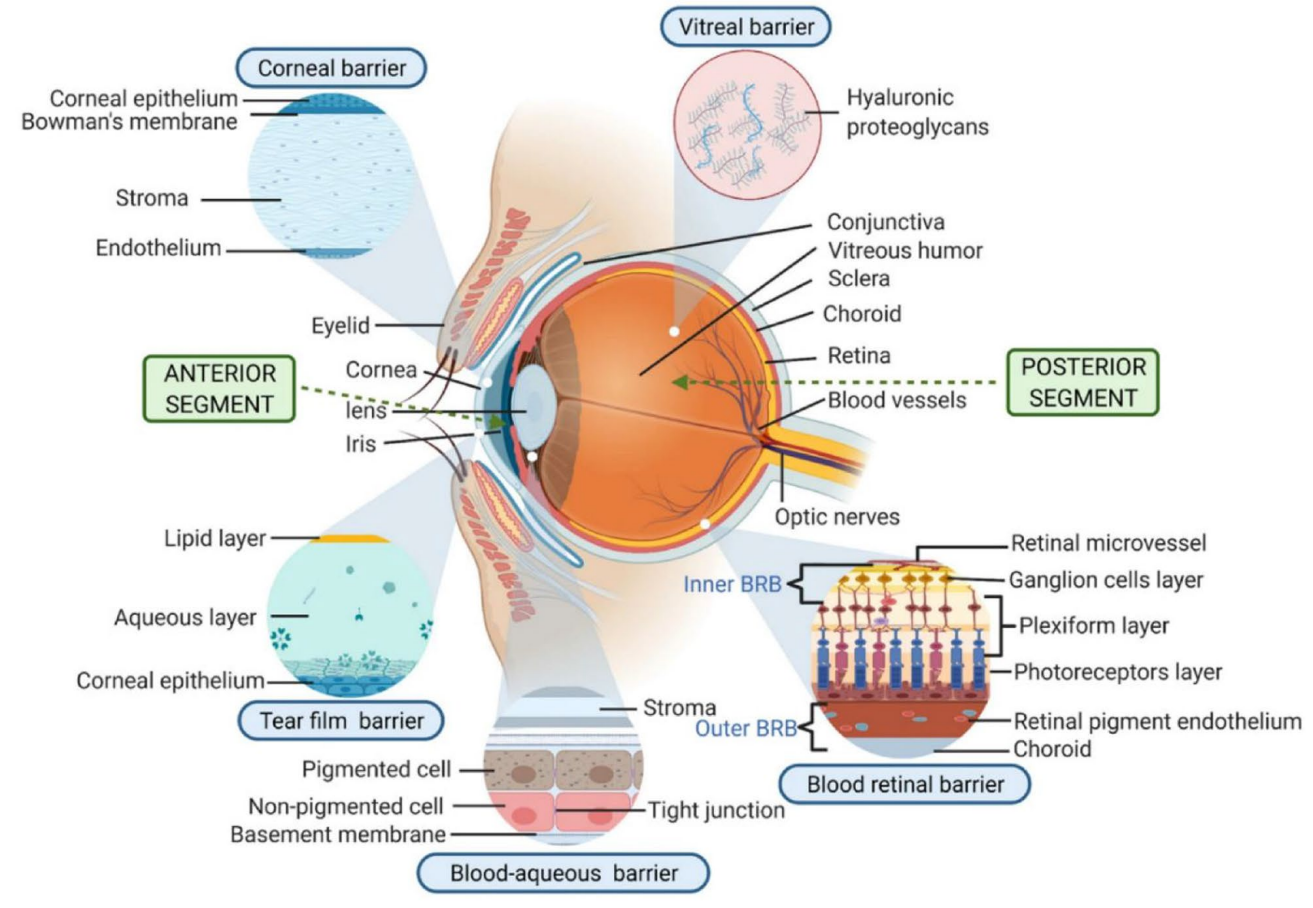
The arrangement of the eye and the barriers involved in delivering drugs through CLs [9]

Glaucoma, which is expected to impact more than 100 million people by 2040, is considered the second leading cause of blindness and is the world’s most common cause of irreversible blindness [1, 2]. The main characteristic of glaucoma is increased intraocular pressure (IOP), leading to damage to the retinal ganglion cells, nerve fiber layer, and optic nerve. While the exact causes of glaucoma are not completely understood, IOP is the only modifiable risk factor in glaucoma management [3]. Consequently, ophthalmic medications that effectively reduce IOP play a crucial role in glaucoma therapy, with the potential to decrease the risk of visual field deterioration by 13–19% for every 1 mmHg reduction in IOP [4, 5]. When it comes to ophthalmic diseases, the systemic administration of drugs is usually ineffective owing to the blood-ocular barrier. Thus, topical administration of ocular drugs is considered the most suitable approach [6]. Nevertheless, the unique physiological and anatomical characteristics of the eye impose several local barriers that restrict the delivery of the drug to the targeted ocular tissues (Fig. 1), posing significant challenges in the treatment of eye diseases [7]. In spite of extensive research studies on improving the effectiveness and overcoming limitations of conventional ocular drug delivery systems for glaucoma therapy, there is still a need for further advancements. Consequently, extensive research has been focused on the development of innovative therapeutic strategies such as contact lenses (CLs) to address the issues associated with the current treatments [8].

In 1965, Sedlácek [10] was the first to use soft CLs as drug delivery system. He soaked the CLs in a 1% homatropine solution, which resulted in greater pupil dilation compared to applying the solution topically. Several studies replicated this method to increase the effectiveness of pilocarpine for the treatment of acute angle closure glaucoma [11–14]. The duration of CLs residence on the ocular surface depends on the exchange between the drug solution and tears. Research by Mcnamara et al. [15] indicated that tear exchange while wearing soft CLs lasted approximately 30 min, in contrast to 5 min without lenses. Preserving the physical properties of polymers is crucial in the fabrication of soft CL as a drug delivery system. The combination of functionalized compounds into hydrogels can impact their water content, oxygen permeability, wettability, flexibility, rigidity, light transmittance, and glass transition temperature [16]. These parameters are important as they influence visual quality and comfort throughout CL wear, thereby affecting the efficacy and safety [17]. It is essential that CLs are designed for drug delivery to meet regulatory standards in terms of acceptable physical properties, comfort, and visual quality to ensure the user’s tolerance [18, 19]. However, it is important to consider the contraindications of wearing CLs, especially in patients with pre-existing ocular conditions [20, 21]. Recent progress in the fields of nanotechnology presents a promising potential for enhancing the duration of drug release from CLs [22]. It is possible to incorporate nanomaterials into the CLs matrix to create a composite drug delivery system, resulting in a prolonged drug release period in comparison to using CLs material alone [23]. Nanomaterial-Loaded CLs offer several advantages over ordinary CLs in disease diagnosis and treatment, including: long-term drug release, increased drug bioavailability, targeted drug delivery, greater tissue penetration, decreased dosing frequency, protection of sensitive drugs, compatibility with different type of drugs, prolonged shelf life, and a well-tolerated and non-invasive experience for patients.

This review focuses on the recent advancements in utilizing nanomaterial-releasing CLs for the diagnosis and treatment of glaucoma. We aimed to investigate the existing glaucoma therapeutics and the limitations associated with current treatment approaches. Additionally, we explored the potential of polymeric materials-laden CLs, lipid materials-laden CLs, and magnetic materials-laden CLs for glaucoma therapy. The review also shed light on the advances made in CLs properties, market perspectives, and the significant challenges faced in the development of therapeutic CLs for ocular drug delivery.

### Current glaucoma therapeutics

Significant advancements have been achieved in the field of glaucoma treatment, providing patients with various options to effectively manage the condition. The three primary classes of treatments currently available are medicated eye drops, laser therapy, and surgical interventions [24].

### Medicated eye drops

Medicated eye drops are one of the most common treatment approaches for glaucoma [25]. These drops aim to lower IOP by either reducing the production of aqueous humor or improving its drainage. Prostaglandin analogs, beta blockers, alpha agonists, and carbonic anhydrase inhibitors are among the different types of eye drops available [25]. These medications are typically prescribed based on the patient’s specific needs, considering the factors such as the severity of the disease, potential side effects, and individual response.

### Laser therapy

Laser therapy, also known as laser trabeculoplasty, is another widely used treatment modality for glaucoma [26]. This procedure involves the use of a laser to enhance the drainage of aqueous by improving the function of the trabecular meshwork. It can be performed in two primary ways: argon laser trabeculoplasty (ALT) and selective laser trabeculoplasty (SLT). ALT uses a nonselective laser to create small burns in the trabecular meshwork, while SLT utilizes a selective laser that targets specific cells, sparing the surrounding tissues [26]. Both procedures aim to reduce IOP and are typically performed in an outpatient setting.

### Surgical interventions

When other treatments fail to adequately control IOP or the patients cannot tolerate medical therapy, surgical interventions may be considered. Trabeculectomy, one of the most common surgical procedures, involves creating a new drainage pathway to bypass the trabecular meshwork, allowing the aqueous humor to drain into the subconjunctival space [27]. Other surgical options include the placement of glaucoma drainage devices, such as a tube shunt, to facilitate the fluid drainage, non-penetrating glaucoma surgeries, and minimally invasive glaucoma surgery (MIGS) techniques, which involve less invasive procedures with quicker recovery times [27].

### Limitations of the current treatments

Although the current treatments for glaucoma have shown to be effective in managing the condition, they do come with certain limitations [28]. Medicated eye drops, for instance, require strict adherence to the prescribed regimen and can potentially cause side effects. Also, due to such factors as rapid clearance, rapid tear turnover, non-productive absorption in the nasal cavity and conjunctiva, and low permeability of the corneal epithelium, the corneal bioavailability of eye drops is typically limited to 1–5% [29, 30]. As a result, high-frequency dosing regimens are often necessary to achieve the desired therapeutic drug concentrations. This frequent dosing can lead to increased side effects and reduced patient compliance [31, 32]. Laser therapy, on the other hand, is not effective for all types of glaucoma and may require repetition over time. Surgical interventions, although generally effective, carry inherent threatening complications, such as hypotonia, infection, bleeding, or cataract formation. Moreover, some patients may not respond adequately to any of the available treatments, necessitating ongoing monitoring and adjustment of the management plan. Consequently, there is a growing body of research which have focused on developing alternative therapies for glaucoma, including the exploration of CL delivery systems.

### Contact lenses for glaucoma management

CLs could be considered as a promising alternative to topical eye drops, which typically have a bioavailability of less than 5%. Unlike eye drops, CLs can be directly placed on the cornea with only the post-lens tear film acting as a barrier [33]. CLs offer several advantages over eye drops. They can retain drugs in the tear film for a longer time, up to 30 min, compared to just two minutes for eye drops. This extended contact time significantly enhances the drug bioavailability by more than 50% [34–37]. Moreover, CLs have additional benefits such as ease of wear, direct interaction with the ocular surface, and their hydrogel composition, and longer contact time, thus having 24-hour effect on IOP. The composition of traditional hydrogel lenses enables the movement of water and nutrients to the cornea, making them suitable for drug delivery approaches involving soaking the lenses in concentrated solutions of active pharmaceuticals [33]. The development of CLs for vision correction has opened up the potential for sustained and controlled drug release in the eye, while maintaining optimal optical performance [36]. CLs are curved plastic disks that adhere to the tear film on the cornea because of the surface tension. They are used not only for vision correction, but also for therapeutic and cosmetic purposes [38]. CLs might be categorized into two main groups: rigid lenses, primarily made of poly(methylmethacrylate) (PMMA), and soft lenses, mostly containing hydroxyethyl methacrylate (pHEMA) polymers [39]. The FDA further categorizes them as hydrophobic or hydrophilic based on their water affinity [40]. Hydrophilic CLs, which have a high-water retention capacity, may be worn for long periods, up to 6 nights and 7 days [41]. Due to the need for improved gas permeability of CLs during closed-eye intervals, silicone CLs were introduced, allowing for continuous wear for 30 days and 29 nights without the ocular hypoxia risk [42, 43]. Since the development of CLs, numerous studies have explained their potential as effective drug delivery systems for treating various acute and chronic eye conditions [44–46]. Therapeutic CLs are typically soft lenses composed of pHEMA polymers, with/without silicone, that are impregnated with drugs using different techniques such as soaking in a drug solution, incorporating drug-releasing colloidal particles, and employing molecular imprinting, ion ligands, or microemulsion-loaded gels [47]. The action mechanism of these systems involves diffusion of the drug in the post-lens tear film, followed by dispersion in the tear fluid and subsequent absorption by the cornea. As the drug continuously gets absorbed in the cornea, the drug concentration in the post-lens tear fluid remains lower than in the CL, resulting in a prolonged flow of the drug from the CL to the cornea. This phenomenon is known as the sink effect [48]. This approach enhances therapeutic efficacy, reduces drug level fluctuations, and lowers the required dosage [49]. Additionally, the use of CLs eliminates the need for preservatives and permeation enhancers usually found in multidose eye drops, which can lead to ocular irritation [50, 51]. These advantages make CLs as viable option for treating chronic ophthalmic conditions, such as glaucoma, that necessitate a consistent therapeutic drug level in the eye.

### Nanomaterial-releasing contact lenses for glaucoma therapy

#### Polymeric materials-laden contact lenses

Polymers show significant promise as potential drug delivery system, owing to their versatile properties including bioavailability, biocompatibility, and robust mechanical properties. Nevertheless, the effective use of polymeric materials in ocular drug delivery requires specialized manipulation or modification due to the unique anatomical structure of the eye. This includes large barriers impeding drug penetration and a rapid drug clearance system that limits drug residence time within the eye. Additionally, the selection of polymeric drug delivery systems should be coordinated with the specific characteristics of the drugs, such as their hydrophobicity and solubility. Recent advances in ophthalmic drug delivery systems employing polymeric materials, especially through CL platforms, have yielded improved results in terms of sustained and long-term drug release profiles. A diverse range of drugs has been successfully incorporated into CLs with polymeric carriers tailored to the physicochemical properties of the drugs. Notably, sustained drug release within the therapeutic range has been substantiated through various in vitro and in vivo approaches [52, 53]. In the following section, recently developed polymeric materials-laden CLs used for glaucoma therapy will be discussed.

**Fig. 2:**
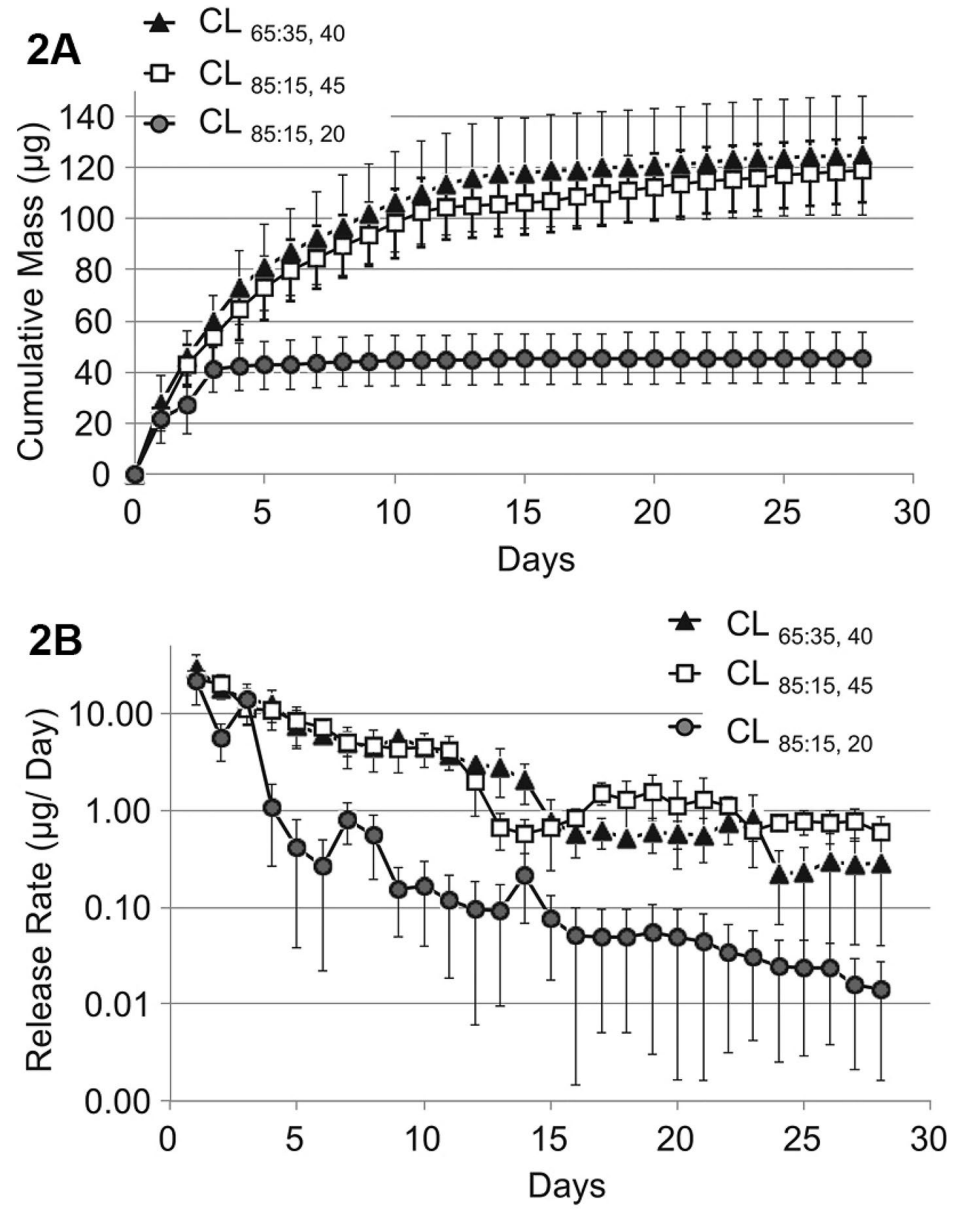
The figures depict the in vitro latanoprost release from CL. Figure **2A** shows the cumulative mass of latanoprost released, while Fig. **2B** presents a semi-log plot illustrating the daily release rate, using the same data as in Fig. **2A**. The data provided represent the average values with standard deviations, and the experiments were conducted four times [54]

Ciolino et al. created CLs containing nanoparticles (NPs) placed within a film layer between the lens material. These lenses utilize the biodegradable polymer PLGA (polylactic glycolic acid) to achieve a sustained release of latanoprost. In vitro and in vivo assays illustrated the capability of this mechanism to consistent delivery of drugs in therapeutic doses over four weeks (Fig. 2). Furthermore, it maintained drug concentrations in the vitreous humor at levels similar to those achieved by traditional eye-drop treatments. Additionally, these lenses proved to be more effective in controlling IOP compared to conventional methods. This observation underscores the ability of PLGA to regulate and prolong drug release for ophthalmic purposes [54].

Xu and colleagues reported a pueranin-cyclodextrin NPs-laden CL for the treatment of glaucoma. Compared with 1% puranin eye drops, CL released a lower amount of the drug (232.64 µg vs. 500 µg). Both the CLs and eye drops exhibited comparable levels of bioavailability in the tear film. Nonetheless, the mean residence time of pueranin in the CLs within the tear fluid of rabbits was approximately 6.04 times greater than 1% pueranin eye drops [55].

Maulvi et al. reported a hydrogel CL containing an ethylcellulose-NPs-laden ring loaded with timolol maleate. They compared this CL with traditional timolol eye drops in rabbits with glaucoma. The group receiving the eye drops experienced a reduction in IOP of 4.4 ± 0.50 mmHg after 2 h, but this effect returned to the baseline levels after 12 h. In contrast, following an early burst release, the CL reduced the IOP by 6.3 ± 1.92 mmHg after 3 h and maintained it below baseline values for 192 h. It was found that the IOP lowering effect was about 1.3 higher, with a lower dose, compared to a single drop and the sustained IOP reduction greater than the in vitro study could be due to the slow release of the drug and low tear volume in the eye (10 µl) in comparison to 2 ml of in vitro release media [56].

In their study, Lee et al. indicated that the addition of lipophilic vitamin E to pHEMA-hydrogel CLs resulted in a significant increase in the loading capacity of hydrophilic substances. They observed a 37.5% increase in the loading of a hydrophilic drug surrogate (Alexa Fluor 488 dye) and a 19.1% increase in the loading of two hydrophilic glaucoma drugs (brimonidine and timolol). Surprisingly, the duration of the drug release was not notably affected by adding vitamin E. Additionally, the researchers investigated the effects of co-loading lipophilic vitamin A onto the lenses and found that it also enhanced the loading capacity of the drug surrogate [57].

In another study by Ciolino et al., the effectiveness of latanoprost-loaded PLGA NPs-laden CLs was compared to latanoprost eye drops in glaucomatous monkeys. Two versions of the latanoprost-loaded CLs were developed, one with a low drug dose and another with a high drug dose. The monkeys were treated consecutively with the low dose CLs for 8 days, followed by latanoprost eye drops for 8 days, and finally the high dose CLs for 8 days. A 3-week washout period was implemented between each treatment. The results indicated that only the high-dose CLs exhibited higher reduction in IOP compared to the eye drops on days 3, 5, and 8. No side effects were observed during the study [58].

Prakash et al. developed NPs-modified drug-loaded biodegradable polymeric CL loaded with a minimal amount of acetazolamide. In an in vitro drug release study using simulated tear fluid (STF), the nano drug complex demonstrated prolonged drug release for 3 h, and the polymer matrix completely degraded within 5 min [59].

**Fig. 3:**
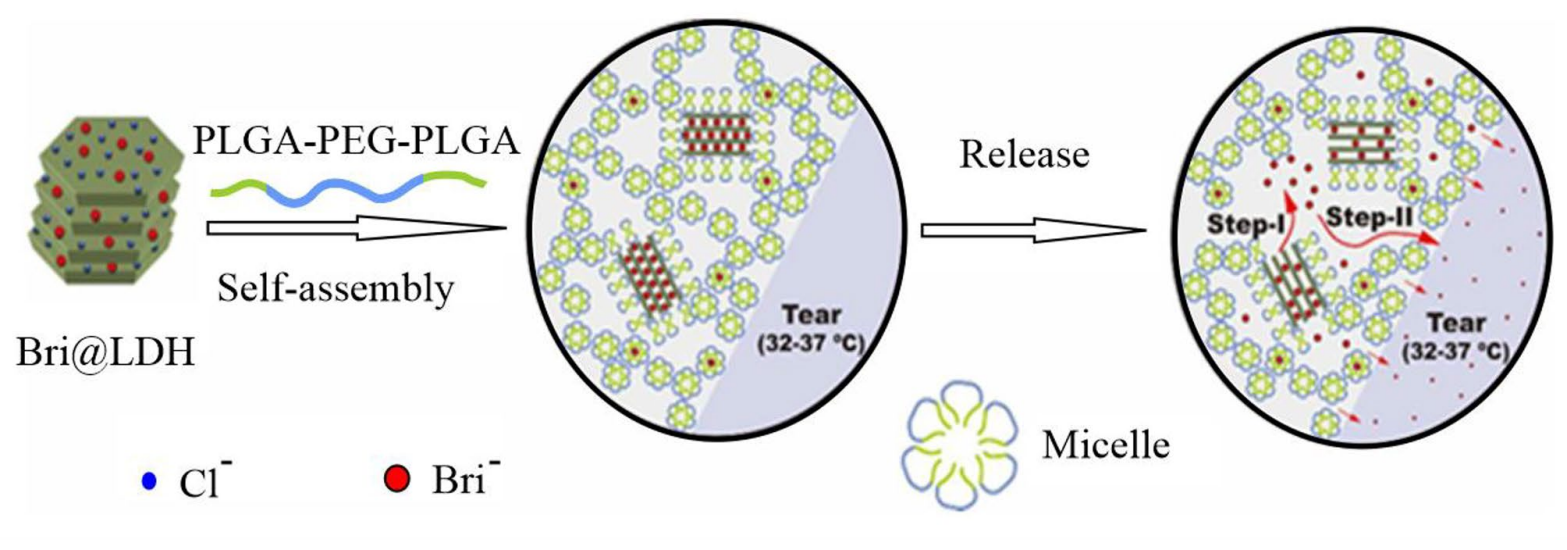
The figure illustrates the formulation approach for the Bri@LDH/Thermogel drug transport system and the dual-regulation approach for drug liberation are visually represented as follows: 1: drug liberation via negative ion substitution from LDH NPs to the thermogel framework; 2: drug diffusion through the thermogel framework to the tear film. Adapted with permission from [60]. Copyright (2023) American Chemical Society

In another study by Sun et al., a novel drug delivery system (DDS) called Bri@LDH/Thermogel was designed for the continuous release of brimonidine. The system involved loading brimonidine onto LDH (Bri@LDH) NPs, which were then dispersed in a thermogel composed of micelles based on a copolymer called poly (dl-lactic acid-co-glycolic acid)-polyethylene glycol-poly (dl-lactic acid-co-glycolic acid) (PLGA-PEG-PLGA). In vitro drug release experiments demonstrated sustained release for up to 144 h, which was a significant delay compared to the release from Bri@LDHNPs alone. Both LDH and thermogel carrier materials exhibited good biocompatibility and were not cytotoxic to human corneal epithelial (HCET) cells. In vivo drug release from the special CL made of Bri@LDH/Thermogel DDS provided sustained brimonidine delivery over 168 h and successfully reduced IOP compared with Alphaganas as a commercial eye drop (Fig. 3) [60].

Mehta et al. developed a novel technique for nano-structured multiple lens coating process. This innovation enhanced the stability of drug formulations on the lens surface and at the bio-interface. The coating process involves the use of polymers such as polyvinylpyrrolidone (PVP) and poly(N-isopropylacrylamide) (PNIPAM), in combination with timolol maleate (TM), to optimize the multi-lens electrohydrodynamic atomization (EHDA) coating procedure. It was found that the coated lenses display a release profile characterized by two phases: an initial burst release followed by a sustained release. Notably, the PNIPAM-coated lenses loaded with TM exhibited the highest drug release, with 89.8% released after 24 h. Permeation studies showed that these coated lenses are well-suited for reducing the dosing regimen, consequently lowering the systemic absorption of the drug. This innovative multiple lens surface coating method has demonstrated the potential for sustained drug release, offering promising outcomes. It introduces a novel approach to enhance the effectiveness of drugs at the ocular surface, ultimately reducing the drug drainage [61].

Lee et al. examined the release of timolol, alone or as loaded in poly (N-isopropylacrylamide) (pNIPAM) nanogels, within the nanoporous structure of bicontinuous microemulsion CLs (BMCLs). They explored different methods of loading the drug and nanogels to understand how temperature affects the release of the drug. Their findings showed that the thermosensitive nanogel-loaded BMCLs released the drug at 35^o^C, while maintaining oxygen permeability and optical transmission. This temperature-triggered drug release near the body temperature has the potential to ensure the controlled release of therapeutic drugs only when the CL is worn, addressing the issue of drug degradation during storage after manufacturing [62].

Sekar et al. investigated the impact of vitamin E incorporation in polymeric hydrogel CLs on the delivery of prostaglandin analogs, specifically bimatoprost and latanoprost. The addition of these nano-sized vitamin E barriers was expected to increase resistance to drug transport and affect the rate of drug release. Initial in vitro experiments showed that ACUVUE OASYS and ACUVUE TruEyeTM CLs, when loaded with 0.2 g of vitamin E/g of hydrogel, significantly extended the release of bimatoprost by 10 to 40-fold. This resulted in sustained delivery of therapeutic doses for more than 10 days. Importantly, the integration of vitamin E did not have a noticeable effect on the transport of latanoprost. Furthermore, an in vivo model predicted more than 50% of corneal bioavailability of bimatoprost using these modified lenses [63].

In a recently published study by Hosseini et al., timolol maleate, an anti-glaucoma drug, was loaded into polymeric NPs composed of chitosan conjugated with lauric acid and sodium alginate. The CLs were surface modified using oxygen plasma irradiation and soaked in varying concentrations of bovine serum albumin (BSA). The drug release from the NPs continued for 3 days, and this duration extended to 6 days after dispersion in the modified lens matrix. By incorporating polymeric NPs and optimizing the surface modification of CLs, this study presents a new approach for sustained drug delivery in the treatment of glaucoma [64].

In the conclusion of this section, we found that the latanoprost-loaded PLGA NPs-laden CL developed by Ciolino et al., is a highly efficient approach for the ocular drug delivery in glaucomatous cynomolgus monkeys. However, studying drug-releasing CLs in monkeys offered many challenges. Due to the smaller ocular surface in the monkey eye, they could retain a smaller volume of eye drops compared to that of humans. Ciolino et al. were able to successfully overcome these challenges. Thus, rather than using a single 50 µl drop that is commonly used for human eyes they administered two separate 25 µl drops into the monkey eyes, with a 5-minute interval. Also, because of smaller ocular surface, they produced CLs with an enhanced base curve and reduced diameter. During the study, no adverse effects were seen.

### Lipid materials-laden contact lenses

Among the various drug delivery systems, lipid-based nanomaterials have the most promising potential. This is primarily attributed to their exceptional biocompatibility, biodegradability, substantial drug-loading capacity, and low immunogenicity [65]. A main advantage of lipid materials-laden CLs is their capability to increase tear film stability. Lipids naturally present in tear help to decrease evaporation and maintain a stable tear film, which is indispensable for eye health and comfort. By integrating lipid components into the lens material, lipid materials-laden CLs can mimic the natural tear film, leading to enhanced comfort, diminished dryness, and extended wearing time. Furthermore, lipid materials-laden CLs have showed a potential for the controlled release of therapeutic agents [66, 67]. In the following section, recently developed lipid materials-laden CLs used for glaucoma therapy will be discussed.

In a study by Li et al. CLs were prepared by incorporating oil-in-water microemulsions (mean particle sizes: 20 to 35 nm) of ethyl butyrate, which were stabilized with the surfactant pluronic F127. The objective was to evaluate the retention capacity and release time of timolol using this approach. It was theorized that the use of the surfactant would enhance resistance to drug release from the lipid vesicles. The findings showed that timolol had a high retention capacity in its basic form. However, the release of timolol was found to be too fast, indicating that pluronic F127 may not have been effective in the extended release of timolol. This could be attributed to the low molecular weight of timolol, which may have hindered the sustained release even in the presence of the surfactant [68].

**Fig. 4:**
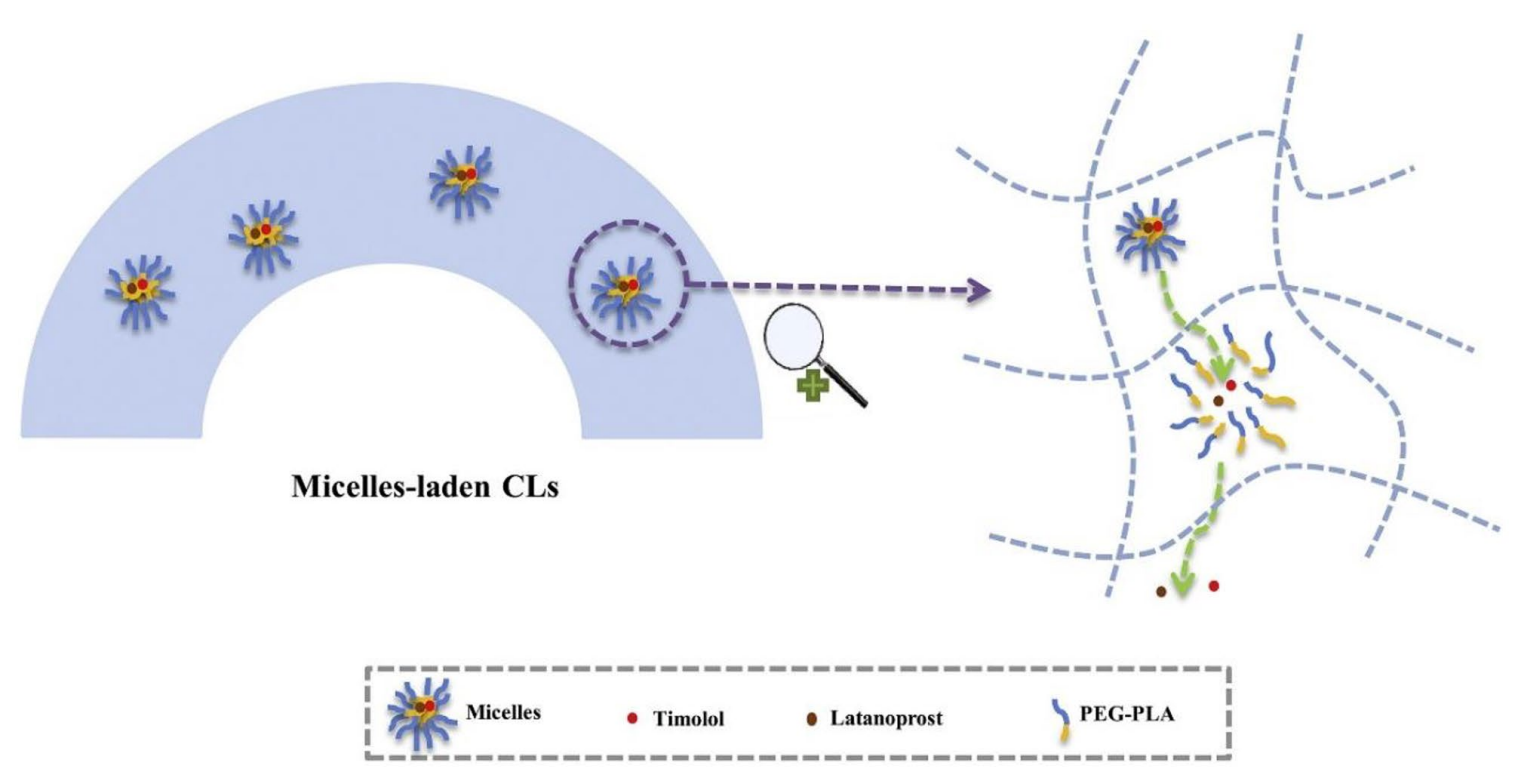
Mechanism of drug release from nanomicelles-releasing CLs. (I) the drug releases from the micelles with the disassociation of micelles, reaching the CLs matrix; (II) the drug subsequently diffuses through the CLs matrix [31]

**Fig. 5:**
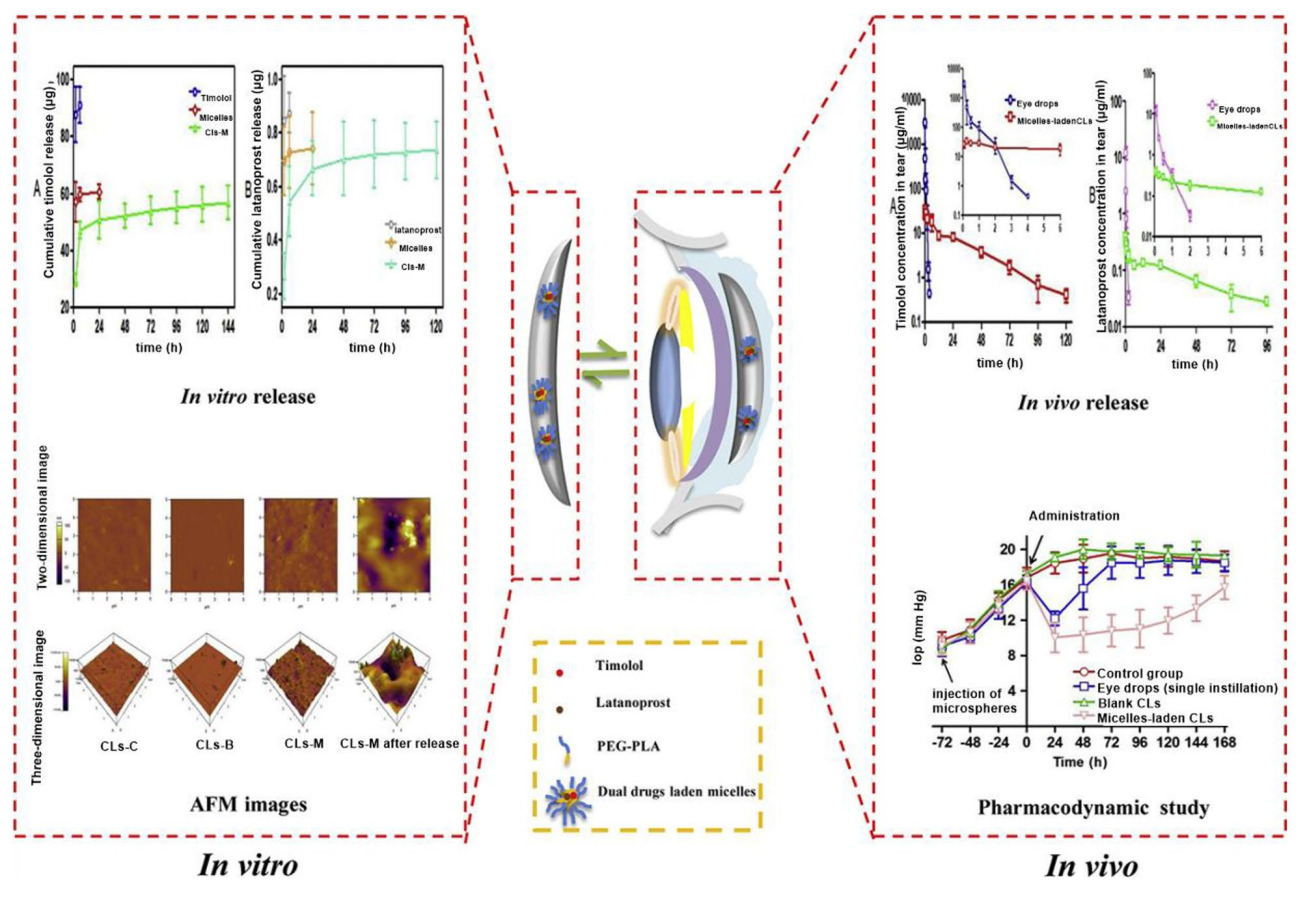
Nanomicelles-releasing CLs (CLs-M) for sustained delivery of timolol and latanoprost [31]

Xu et al. reported nanomicelles-releasing CLs (CLs-M) designed for sustained delivery of timolol and latanoprost simultaneously, aiming to enhance the patient compliance and achieve the desired IOP lowering effect. The release of latanoprost and timolol from CLs-M occurred gradually over 120–144 h. The drugs were believed to diffuse through the micelles, reach the CLs matrix, and subsequently diffuse through the matrix into the release media (Fig. 4). In vivo pharmacokinetic (PK) studies conducted on rabbit eyes demonstrated sustained release of timolol for up to 120 h and latanoprost for up to 96 h in the tear film. Notably, CLs-M exhibited significantly improved mean bioavailability (2.2-fold and 7.3-fold) and residence time (79.6-fold and 122.2-fold) compared to eye drops for both timolol and latanoprost respectively. In an in vivo pharmacodynamic (PD) study using a rabbit model with elevated IOP, sustained IOP reduction was observed for over 168 h. Figure 5 depicts the comprehensive in vitro release profile of latanoprost and timolol in combination. The micelles initially displayed a high burst release of timolol (57.12 µg) and latanoprost (0.70 µg) with no detectable levels of the drugs after 24 h. In contrast, CLs-M exhibited a relatively lower burst release of 29.68 µg of timolol and 0.27 µg of latanoprost, with the release duration extending up to 144 h and 120 h, respectively [31].

Xu et al. conducted a study to examine the impact of microemulsion on bimatoprost uptake from the soaking solution and its effect on drug release kinetics in CLs. The CLs were soaked in a bimatoprost-microemulsion (average size: 25.65 to 39.69 nm) soaking solution (ME) and compared to a bimatoprost-soaking solution (SM) without microemulsion. The results revealed a two-fold increase in the uptake/loading of bimatoprost from the ME soaking solution compared to the SM solution. This indicates that the microemulsion facilitated enhanced uptake of bimatoprost by the CLs. In terms of drug release kinetics, in vitro release profiles of the ME CLs demonstrated improved release rate profiles, with sustained release observed for up to 96 h. On the other hand, the SM CLs exhibited release for up to 48 h based on the in vitro data. In in vivo studies conducted on rabbit tear fluid, the ME CLs showed low burst release and improved retention of bimatoprost compared to the SM CLs and eye drop solution. This suggests that the incorporation of microemulsion into the soaking solution contributes to a controlled release of bimatoprost and enhanced drug retention in the CLs [66].

Xu et al. reported a study to demonstrate the application of a microemulsion system for improved loading of travoprost in CLs and assess its impact on the swelling and optical characteristics of the lenses. The travoprost-loaded microemulsion (average size: 21.11 to 43.85 nm) soaked CLs (T-ME) were compared to conventional CLs soaked in a travoprost-packaging solution (T-SM). The results showed that the T-ME CLs exhibited enhanced drug uptake (loading) compared to T-SM CLs. Additionally, the physical characteristics of the CLs, including swelling and optical properties, were improved when using the microemulsion system. The in vitro release data (flux data) demonstrated a slower release profile for the T-ME CLs, with release occurring over 48–120 h. In comparison, the T-SM CLs exhibited a faster release profile, with release occurring over 36–48 h. In an in vivo drug release study conducted on the tear fluid of New Zealand rabbits, T-ME CLs exhibited a greater drug retention compared to the eye drop solution [69].

Wei et al. conducted a study to examine the impact of microemulsion on the loading and release kinetics of timolol from soaking solutions in CLs. The CLs were soaked in a timolol-microemulsion (The average size: 38.53 to 73.76 nm) soaking solution (TB-MESM) and compared to a timolol-soaking solution (TB-SM) without microemulsion. The incorporation of timolol-releasing microemulsion into the CLs did not affect their transmittance properties and swelling. The results showed a two-fold improvement in the loading of timolol from the TB-ME-SM soaking solution compared to the TB-SM solution. The release rate profiles of the TB-ME-SM lenses were also improved, with sustained release observed for up to 48–96 h, whereas the TB-SM lenses exhibited release for up to 24–36 h based on flux data. The rabbit tear fluid analysis demonstrated improved retention time with the TB-ME-SM lenses compared to the TB-SM lenses and eye drop solution. In an efficacy study conducted on a rabbit model, the TB-ME-SM-2 CLs exhibited a prolonged reduction in IOP for 96 h, while eye drop therapy showed a peak and valley profile [70].

A novel approach involved the development of latanoprost-loaded PEGylated solid lipid NPs (LP-pSLNs) to improve the loading capacity of latanoprost in CLs (LP-pSLN-L) and sustained ocular drug delivery. Comparison of different lens formulations showed that, the LP-SM-L lens exhibited low drug loading, high burst release, and short release duration of 24 h. In contrast, the LP-SLN-L and LP-pSLN-L lenses demonstrated high drug uptake and sustained drug release for up to 120 h and 96 h, respectively. The incorporation of PEG diminished the size of the NPs and enhanced the capacity of drug loading in the lenses. However, the release rate was initially high in the first few hours. In animal studies, the LP-pSLN-10-L batch exhibited a high drug up to 96 h when compared to the LP-SM-L lens and eye drop solution [71].

In conclusion, among all lipid materials-laden CLs discussed in this section, the authors think that nanomicelles-releasing CLs designed by Xu et al. [31] provide a promising approach for the co-delivery of timolol and latanoprost in patients with glaucoma. In this study, they successfully overcame the challenge associated with NPs-laden CLs, which have low transparency thereby restricting their potential applications. For maintaining transparency, micelles thanks to their smaller particle size, prove to be a more suitable option for incorporating into CLs [31]. In this regard, Chauhan et al. showed that HEMA hydrogels containing cyclosporine A micelles did not sacrifice optical properties and provided sustained drug release [72]. In addition, hydrophilic groups on the surface of micelles are more compatible with the CL matrix than the hydrophobic surface of polymeric NPs [73].

### Magnetic materials-laden contact lenses

Magnetic nanomaterials stand out from the other nanocarriers because of their magnetic properties making them unique for drug delivery purposes [74]. Drug molecules might attach to their shell to inter the body and be concentrated in specific areas through the impact of an external magnetic field facilitating the controlled migration and enhancing the effectiveness of treatment. Because of the remarkable surface-to-volume ratio, it offers numerous chemically active sites for binding biomolecules [7]. Magnetic NPs can be loaded with therapeutic agents and embedded into the lens material, enabling localized drug release to the ocular surface (7, 74). In the following section, recently developed magnetic material-laden CLs used for glaucoma therapy will be discussed [7, 75].

**Fig. 6:**
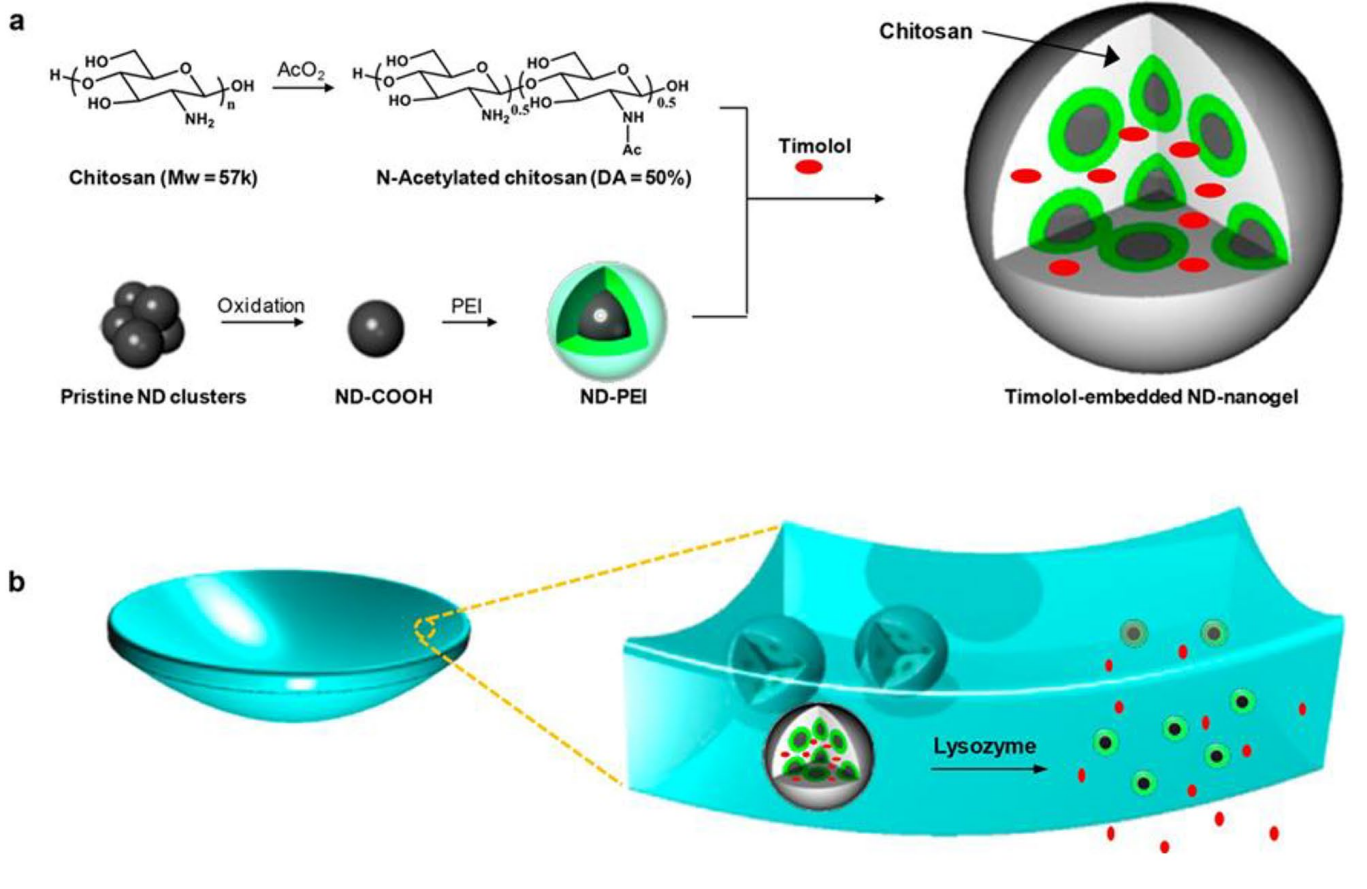
The lysozyme-activated drug-eluting CL is illustrated as follows: (**a**) The process begins by synthesizing drug-loaded ND-nanogels through the cross-linking of PEI-coated NDs (nanodiamonds) and partially N-acetylated chitosan in the presence of timolol maleate. These ND-nanogels containing the drug are then embedded in a hydrogel and cast into CLs that are responsive to enzymes. (**b**) When exposed to the lacrimal fluid containing lysozyme, the N-acetylated chitosan present in the ND-nanogels is cleaved by the enzyme. This enzymatic cleavage leads to the degradation of the ND-nanogels, resulting in the release of the encapsulated timolol maleate. Importantly, the CLs remain intact throughout this process. This schematic illustration demonstrates the mechanism by which the lysozyme-activated drug-eluting CL operates, where the presence of lysozyme triggers the controlled release of the drug while maintaining the integrity of the lens [76]

Kim et al. have conducted a study to develop a nanodiamond (ND)-embedded CLs that enables the release of timolol maleate (TM) triggered by lysozyme. The researchers utilized enzyme-cleavable polymers in the ND-embedded lenses to achieve controlled release of TM in the presence of lysozyme. The study confirmed the retention of drug activity in primary human trabecular meshwork cells, demonstrating the effectiveness of the ND-embedded lens in activating drug release in the presence of lysozyme. These findings highlight the potential of the ND-embedded lens as a promising approach for sustained drug delivery in the management of glaucoma. The controlled release mechanism, triggered by the presence of lysozyme, offers translational benefits in providing effective therapy and maintaining drug activity over a prolonged time period (Fig. 6) [76].

Maulvi and colleagues conducted a study to develop a formulation by gold NPs (Au-NP) loaded with timolol for glaucoma treatment. Two methods were employed to incorporate timolol into the Au-NP. One approach involved soaking Au-NP in a timolol solution, while the other method combined Au-NP with CLs. The study evaluated the properties of the CLs, such as swelling and optical transmittance, after loading them with Au-NP. It was found that these properties remained unchanged, indicating that the presence of Au-NP did not affect the physical characteristics of the lenses. In vivo pharmacokinetic and pharmacodynamic studies were conducted to compare the prepared formulation with conventional methods in terms of timolol concentration. The results demonstrated that the formulation with Au-NP led to a significant decrease in IOP (72 h) because of the large amounts of drug delivered to the favorite site [77].

Li et al. (2021) conducted a research to investigate the IOP lowering effect of bimatoprost either loaded by a conventional soaking method or by adding GNP solutions into the bimatoprost soaking solution [78]. Initially, bimatoprost exhibited a poor drug uptake, high burst release, and compromised lens properties when compared to the traditional solution. Subsequently, the researchers examined the impact of GNP and their release kinetics. The CLs were infused with 21.1 nm GNP [53, 78]. By incorporating GNP, the oxygen permeability and optical transmittance of the CLs increased after soaking it in the solution. In vitro studies demonstrated a substantial enhancement in the sustained release of the drug, extending the release time from 24 to 36 h, with a reduced burst release rate. Additionally, the presence of GNP contributed to the reduction of protein adherence [79].

Kumar et al. developed levobunolol-loaded Eudragit-based NPs incorporated into a CL to achieve sustained ocular delivery of the drug. They evaluated the equilibrium swelling index and the transmittance of the CL incorporated with the NPs, and compared them to lenses loaded with a solution of the drug. The results demonstrated improved swelling index and transmittance in the lenses with NPs incorporation. In vitro release studies were conducted to assess the drug release profiles from the CL. The findings showed more sustained drug release patterns from the lenses loaded with NPs compared to lenses those loaded with a drug solution. Furthermore, ex vivo transcorneal permeation studies were carried out to examine the permeation of the drug through the CL. The results showed enhanced permeation through the CLs compared to commercially available eye drops [80].

Maulvi et al. performed a study to investigate the effects of nano graphene oxide (GO) on the uptake, swelling properties, transmittance, and release of bimatoprost in CLs. The researchers examined whether the presence of GO could enhance the uptake of bimatoprost, and whether it would affect the transmittance and swelling properties of the lenses. They also investigated whether the incorporation of GO could lead to sustained release of bimatoprost. The results showed that the presence of GO enhanced the swelling of the CLs because of its water-binding capacity. Additionally, the transmittance of the lenses was enhanced by the molecular dispersion of bimatoprost on the GO surface; this prevented the local precipitation of the drug. Although the uptake of bimatoprost was not improved in the presence of GO, the in vitro release profile of the drug was enhanced. Increasing the amount of GO resulted in a substantial decrease in the cumulative and burst release of bimatoprost from the lenses. In vivo pharmacokinetic studies in rabbit tear fluid demonstrated a substantial improvement in the area under the curve (AUC) and mean residence time (MRT) with the DL-GO-0.2 µg-BMT-100 CL, compared to the eye drop solution [81].

In a recently published study, GO as a single monomolecular layer nano-sheets, was integrated into CLs during the production process to control the release profile of timolol and improve the physical and optical properties of the lenses. The researchers compared GO-releasing CLs soaked in a timolol solution (SM-GO-TB) with blank CLs (SM-TB) soaked in the same solution. They also fabricated direct timolol-GO-lenses (DL-TB-GO) and direct timolol-lenses (DL-TB) by adding timolol-GO and timolol during the polymerization process, respectively. The presence of GO in both soaked and directly releasing CLs resulted in improved swelling properties, and this was attributed to the formation of hydrogen bonds between GO and water molecules. Additionally, the transmittance of the GO-releasing CLs was considerably improved because of the molecular dispersion of the drug on the GO surface. GO incorporation in the direct and soaked timolol-releasing CLs led to a significant reduction in burst release and improved release profiles compared to the respective CLs without GO. This suggests that GO can effectively control the release of timolol and enhance the sustained delivery of the drug [82].

Fan et al. developed an innovative CL by incorporating silica-based pH-responsive nanocomposite particles that allow the controlled release of timolol maleate under physiological pH conditions. It was found that silica-alginate composite NPs did not exhibit significant pH-responsive release of timolol maleate. However, silica-poly (methacrylic acid) NPs showed promising pH-responsive release behavior. These NPs demonstrated an ON/OFF triggered release mechanism, where only a fraction of the drug was released at low pH (pH 2.5), and then sustained release occurred at physiological pH. The release profiles of timolol maleate from the silica-poly (methacrylic acid) NPs demonstrated a controlled and sustained release pattern, with less than 15% of the drug released under acidic conditions and a long-lasting release in a simulated tear fluid, representing the physiological pH conditions [83].

A novel approach was developed by incorporating brimonidine-loaded silica NPs into silicone CLs (Bri-Si) to reach controlled drug delivery while preserving the optophysical properties of the lenses. The direct brimonidine-loading method (Bri-DL), traditional soaking method (Bri-SM), and microemulsion-releasing CL (Bri-ME) were also developed for comparison. The Bri-Si lens exhibited improved oxygen permeability, transmittance, swelling, and reduced lysozyme adherence compared to the Bri-SM, Bri-DL, and Bri-ME lenses. The Bri-DL lens exhibited high leaching of brimonidine during extraction and sterilization, resulting in low cumulative drug release. On the other hand, the Bri-Si lens demonstrated controlled release of brimonidine for up to 144 h. In a rabbit tear fluid model, the Bri-Si lens maintained a high concentration of brimonidine for 96 h and performed better than the Bri-ME lens and eye drop therapy [84].

In a study by Lai et al., pH-triggered drug-containing lenses (DCLNs) combined with large-pore mesoporous silica NPs (LPMSNs) were investigated for the sustained release of glaucoma drugs. The LPMSN-releasing DCLNs showed improved drug retention in an artificial lacrimal fluid (ALF) environment at pH 7.4 compared to reference DCLNs. Unlike reference DCLNs, LPMSN-releasing DCLNs do not need preloading of the drug and can be easily integrated into the current CL fabrication processes. LPMSN-releasing DCLNs soaked at pH 6.5 demonstrated enhanced drug loading capacity compared to reference DCLNs due to specific adsorption onto the LPMSNs. The sustained release of glaucoma drugs from LPMSN-releasing DCLNs was observed and monitored in the ALF, providing controlled drug release over time. The cytotoxicity of LPMSN-releasing DCLNs was evaluated, and quantitative and qualitative results indicated no cytotoxic effects. This suggests that LPMSN-releasing DCLNs are safe for ocular use [85].

An overview of the reported magnetic material-laden CLs for the glaucoma therapy revealed that the nanodiamond (ND)-embedded CLs designed by Kim et al. can be a highly promising approach for sustained drug delivery in the glaucoma therapy. In this study, they overcame the major challenge of premature removal of the drug and inefficient drug delivery system of CLs by developing a smarter delivery system with lysozyme-triggered drug delivery that enabled the controlled drug release. Also, integration of nanodiamonds (NDs) into the lens material has resulted in the creation of more durable lenses without sacrificing the lens thickness or user comfort. This overcame the challenge of low oxygen permeability and mechanical strength of the lens.

### Nanomaterial releasing contact lenses for diagnosis of glaucoma

Nanomaterials represents potentially significant changes in the strategies used for diagnosis and imaging, offering a profound insight into the underlying molecular mechanisms of diseases. These advances have the potential to revolutionize medical practices by enabling more customized treatment approaches, ultimately leading to improved results for patient [86]. Nanomaterial-releasing CLs have developed as a hopeful device for glaucoma diagnosis, presenting innovative solutions to increase detecting and monitoring this sight-threatening condition. These CLs are constructed using embedded nanomaterials that enables specific and sensitive IOP measurements, a significant parameter for glaucoma diagnosis. The nanomaterials incorporation, such as NPs or nanosensors, into CLs lets for real-time IOP monitoring, offering non-invasive and continuous measurements. These nanomaterials are capable of exhibiting exceptional properties, such as electrical or optical responses to pressure changes, enabling sensitive and accurate detection of IOP variations associated with glaucoma [87–89].

Kim et al. developed a smart CL that incorporates a strain sensor of the serpentine silicon nanomembrane to monitor IOP. The strain sensor is embedded within the lens and can accurately measure IOP. The CL also includes an antenna for transmitting the measured IOP values. The effectiveness of the smart CL in IOP monitoring was demonstrated by comparing the results obtained with those from a tonometer. This study specifically focused on monitoring IOP in diabetic peoples who had undergone intraocular islet transplantation, showcasing the potential of the smart CL for IOP monitoring in clinical setting [90].

In another study by Fan et al., a piezo-resistive pressure sensor was developed to non-invasively and continuously monitor IOP. The sensor utilized a Wheatstone bridge circuit and was fabricated using a spray-coating method. The sensing layers of the sensor consisted of hybrid nanomaterials, namely graphene and carbon nanotubes, which were embedded within a soft CL substrate composed of flexible polydimethyl siloxane (PDMS) and parylene. The sensor demonstrated high sensitivity (36.01 µV mmHg^−1) based on the tests conducted on a PDMS eyeball model. The sensor also exhibited good frequency response and the capability to track dynamic pressure changes within the normal IOP range of 9 to 34 mmHg. It displayed good repeatability, linearity, and accuracy in tracking fluctuating IOP levels [91].

Liu et al. created a non-invasive technique for ongoing IOP monitoring by utilizing a novel strain gauge material composed of graphene nanowalls. They investigated the relationship between the corneal strain, CLs, and IOP through experimentation. To enable the non-invasive IOP monitoring, a new approach was developed to transfer graphene nanowalls onto a CL using a gold film. The sensitivity of this device for detecting IOP was found to be 42,250 ppm/mmHg, surpassing that of the IOP measured by tonometer [92].

Kim and colleagues have developed a soft nano-based CL capable of real-time IOP monitoring in human eyes. The lens utilizes a cellphone for the diagnosis of glaucoma progression. For measuring IOP, a strain sensor is positioned in the elastic zone where strain is concentrated, employing the modulation of Young’s modulus. The lens integrates electronic circuits for near-field communication (NFC) with stretchable and high-resolution interconnections, enabling wireless and battery-free operation. Through human pilot trials and in vivo studies on live rabbits, the lens demonstrated excellent biocompatibility, achieving accuracy comparable to the gold-standard tonometry. Additionally, the lens exhibited stability against inflammation, thermal exposure, and electromagnetic radiation, while causing minimal corneal abrasion [93].

Researchers have developed a CL sensor utilizing self-assembly graphene (SAG) for continuous IOP monitoring. By combining face-to-face water transfer printing and micro-electromechanical systems technology, they achieved batch preparation of the sensor. The sensor demonstrated excellent temperature stability and light transmittance. Used on a silicone eye, it showed an ultra-high sensitivity to IOP, measuring 1.0164 mV mm Hg−1. In vitro testing on a porcine eye showed a sensitivity of 3.166 mV mm Hg−1 with notable linearity. The sensor sensitivity is sufficient to be read by a commercial radio frequency identification read write/system, enabling continuous wireless IOP monitoring [94].

Kim and colleagues have reported a smart CL that incorporates a transparent silver nanowire IOP strain sensor and wireless circuits, enabling continuous and noninvasive IOP monitoring. The stability of the IOP sensor within the smart CL was verified in the presence of tears and through repeated eyelid blink model cycles. In vitro tests were conducted on polydimethylsiloxane model eyes, demonstrating the ability to monitor changes in IOP. Furthermore, in vivo experiments on live rabbit eyes confirmed successful monitoring of IOP changes by the fully integrated wireless smart CL, which was confirmed against the conventional invasive tonometer IOP test. These findings demonstrated the possibility of the smart CL as a noninvasive platform for continuous IOP monitoring in glaucoma patients [95].

**Fig. 7:**
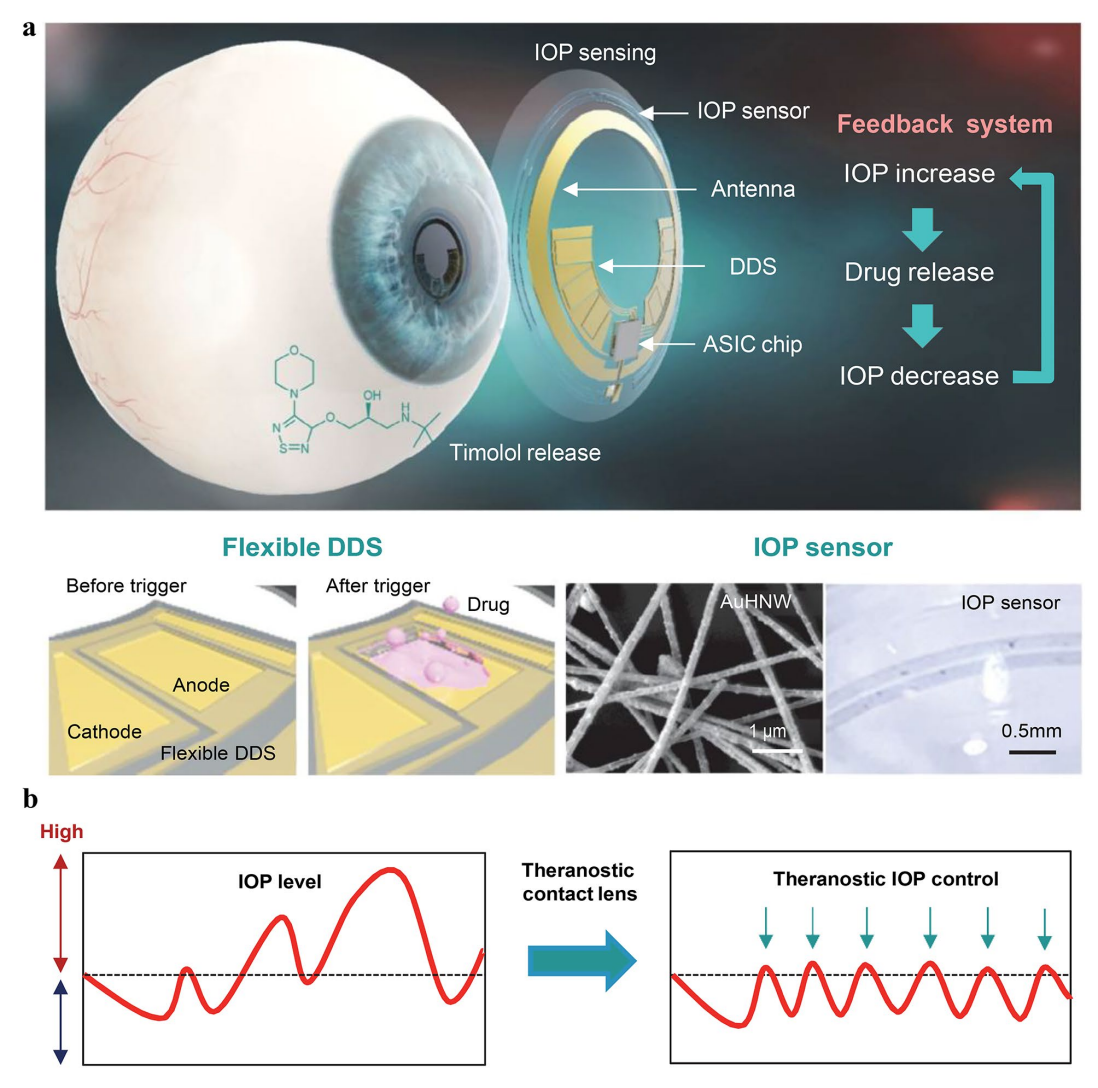
A theranostic smart CL for management of glaucoma is as follows: (**a**) The theranostic smart CL is depicted, showcasing its structure that includes a fully integrated IOP sensor based on gold hollow nanowires (AuHNW), a DDS, and wireless circuits. This integrated system enables wireless glaucoma treatments with a feedback mechanism for IOP sensing and controlled release of timolol. (**b**) A schematic representation compares the conventional continuous IOP monitoring with the IOP control achieved through IOP monitoring and on-demand drug delivery. This approach allows for glaucoma management [96]

Kim et al. have introduced a very integrated theranostic smart CL for IOP monitoring in glaucoma. This lens included a flexible DDS, a sensitive IOP sensor based on gold hollow nanowires (AuHNW), wireless power and communication systems, and an application-specific integrated circuit (ASIC) chip. The AuHNW-based IOP sensor exhibited excellent ocular strain sensitivity, biocompatibility, and chemical stability. Additionally, the flexible DDS enabled on-demand delivery of timolol to lower the IOP. Overall, the theranostic smart CL successfully monitord the IOP levels in rabbits with glaucoma, as demonstrated in the study (Fig. 7) [96].

**Fig. 8:**
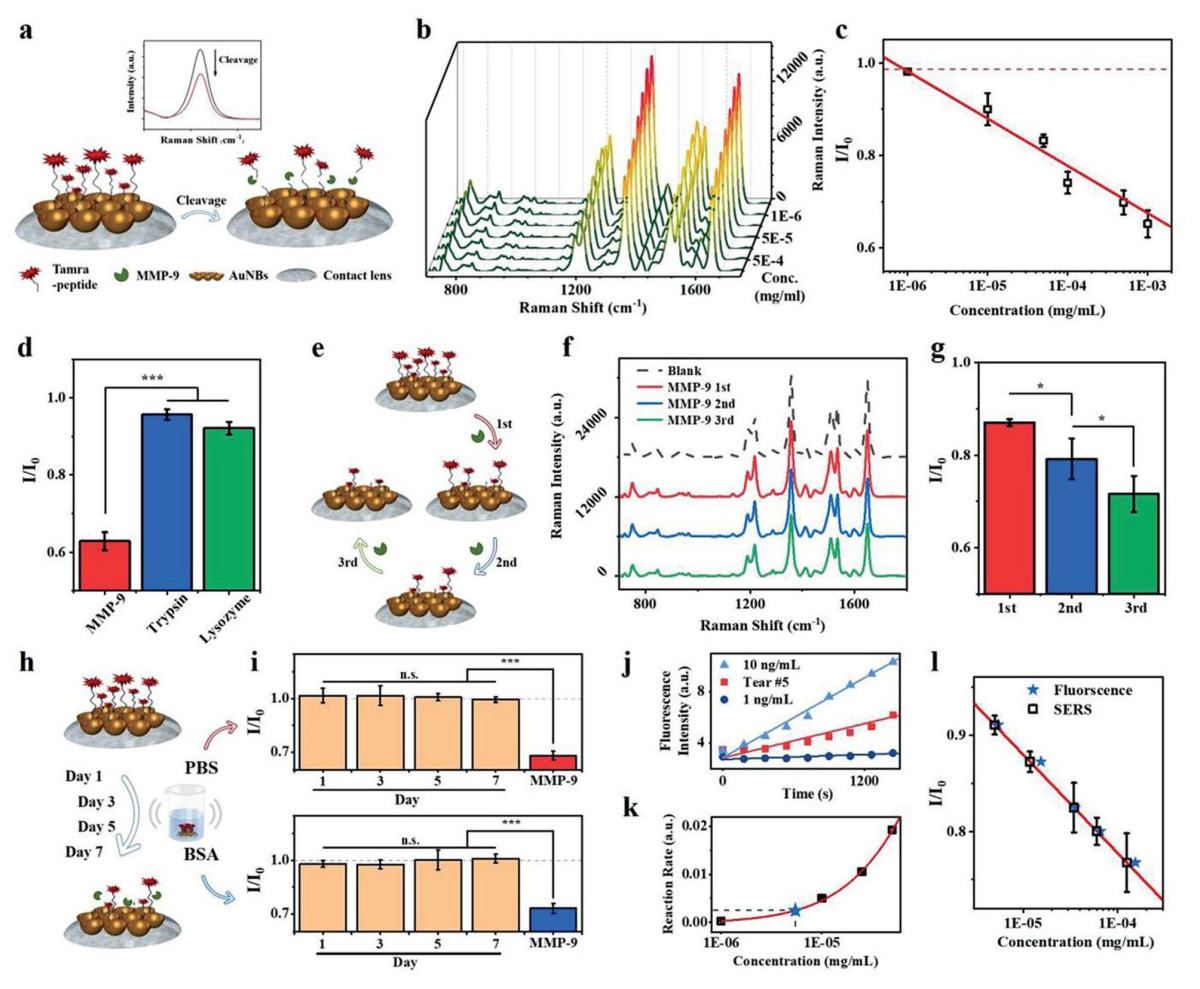
(**a**) Cleavage of Tamra-pep by MMP-9 on SERS substrate. (**b**) Raman spectra of Tamra-pep on AuNBs after MMP-9 treatment. (**c**) Linear calibration curve for Raman intensity ratios and MMP-9 concentration. (**d**) SERS responses to MMP-9, trypsin, and lysozyme. (**e**) Repetitive MMP-9 detection on CLs. (**f**) Raman spectra after MMP-9 incubation. (**g**) SERS responses after multiple MMP-9 treatments. (**h**) Stability test over seven days. (**i**) SERS intensity ratios over time with MMP-9 cleavage. (**j**) Fluorescence kinetics of MMP-9 and Tear #5. k) MMP-9 level on fluorescent assay calibration curve. l) Comparison of measured and calculated MMP-9 concentrations [97]

A dual-functional smart CL sensor utilizing optical-based technology has been developed for IOP monitoring and detecting matrix metalloproteinase-9 (MMP-9), which is an important biomarker in glaucoma. The sensor incorporates an antiopal structure that undergoes color change in response to IOP elevation with no need for complex electronics. Additionally, the sensor includes peptide-modified gold nanobowls (AuNBs) as a surface-enhanced Raman scattering (SERS) substrate, enabling the quantitative analysis of MMP-9 at low nanomolar concentrations in real tear samples. These dual-sensing functions demonstrated a noninvasive, convenient, and possibly multifunctional platform for monitoring diagnostic biomarkers in human tears. Figure 8 provides an illustration of the concept [97].

Regarding all the nanomaterial-releasing CLs reported for diagnosis of glaucoma in this section, it seems that the diagnostic system reported by Kim et al. [96] holds significant promise for IOP monitoring in the human eye. As previous studies have shown, smart CLs suffer from limited sensitivity, poor stability, and low biocompatibility for long-term IOP monitoring. To address these issues, Kim et al. synthesized AuHNW (gold hollow nanowire) that offered high sensitivity, excellent biocompatibility and stability with relatively high transparency for long-term IOP monitoring and achieved an accuracy comparable to gold standard tonometry. Additionally, they designed a flexible DDS with a biocompatible protective layer for high drug-loading efficiency into a smart CL.

### Improving the properties of contact lenses

Optimization of CLs properties for glaucoma management includes some key considerations to ensure their efficacy. These optimization attempts focus on several facets, including the long-term wear ability, comfort, IOP measurement, and drug delivery [98]. Optimizing CLs for IOP measurement contains the incorporation of precise and reliable sensors or measurement methods. Advances in sensor technologies, such as nanosensors, allow real-time IOP monitoring [99]. The sensitivity, specificity and accuracy of the sensors are very important to enable early diagnosis of glaucoma and timely intervention [98]. CLs designed for drug delivery require to be optimized to ensure sustained and controlled release of medications [8]. This includes choosing suitable drug-loaded carriers, such as hydrogel matrices, that can release the medication in a controlled manner over a long period. The CLs design must assist effective drug loading/release, maintaining therapeutic drug levels while minimizing adverse effects. Comfort is a critical issue in optimizing CLs for glaucoma patients. Improving the biocompatibility and surface properties of the lenses can minimize inflammation, irritation, and discomfort [100]. Surface modifications, such as incorporating hydrophilic coatings or decreasing protein adsorption, can improve the wearability and comfort of the lenses, ensuring long-term usage and patient adherence [101]. Moreover, optimization of the longevity and durability of CLs is significant to support their clinical application in glaucoma management. The CLs must be able to withstand the rigors of daily wear, maintain optical properties and their shape, and resist protein or lipid deposition. This may be done via material selection, design improvements, and suitable manufacturing approach. Finally, clinical trials and investigations are essential to assess the efficacy, safety, and performance of the optimized CLs. These trials must involve glaucoma patients and evaluate parameters such as IOP measurement accuracy, drug release profiles, patient comfort, and long-term ocular health outcomes [52]. In summary, by developing sensor technologies, surface modifications, DDSs, and accurate clinical evaluation, we can optimize CLs to successfully manage glaucoma and enhance the patients’ quality of life.

### Marketing perspectives

The global market for therapeutic CLs is experiencing significant growth. Key companies competing in this market include UltraVision CLPL, Johnson and Johnson Vision Care Inc., Unilens Corporation, Bausch & Lomb Incorporated, Vistacom Inc., and Alcon Pharmaceuticals Ltd. [102, 103]. Although many of these products are still in the preclinical/clinical study stages [104], the growing interest in therapeutic CLs is driven by various factors. These include the aging population, increasing prevalence of eye disorders like glaucoma, rising number of vision correction surgeries and cataract procedures that require post-operative treatments, and the potential to increase the re-epithelialization rate of ocular tissues. Additionally, the need for therapeutic CLs is boosted by their capability to diminish patient discomfort. In selecting therapeutic CLs, the choice mainly depends on the specific pathology. Nevertheless, there are some essential prerequisites and current challenges that therapeutic CLs must meet, including high oxygen permeability and cost-effectiveness [103]. The range of parameters for therapeutic CLs includes the total diameter (TD) and back-optic zone radius (BOZR). Standard TDs are typically used for soft lenses, but in certain cases, larger TDs may be required, for example for preventing post-operation wound leak. For a proper physical fit, CLs with larger TDs necessitate a flatter BOZR. The CL stability on the eye relies on minimizing hydrogel dehydration that may occur following the use of lens. However, this poses a challenge for patients with dry eye. To minimize impurity deposition on the lens surface, it is ideal for the lens material to be resistant to such formation. While disposable lenses are a practical option, they may reduce the patient compliance, and therapeutic effectiveness. Regulatory considerations also come into play for the marketing of therapeutic CLs. Determining whether the lenses are classified as drugs or combination products is a primary regulatory aspect. If the lens is solely a support for ocular drug delivery, it would probably be classified as a drug. However, if the CL also serves as a device with additional functions like refraction correction, it may be regarded as a combination product. For development of a novel ophthalmic drug delivery platform, a common method is loading drug molecules previously approved by the US FDA. However, additional preclinical and clinical investigations may be needed to ensure the pharmacokinetics, efficacy, and safety of the new product [103].

### Major challenges of diagnostic and therapeutic contact lenses

Therapeutic CLs for ophthalmic drug delivery face some main challenges that need to be addressed for their successful progress and widespread use. Achieving the desired capacity of drug loading and controlled release profile is a significant challenge [8, 85, 105]. It includes development of appropriate DDSs that can successfully load the drugs and release them in a controlled manner [106–108]. The challenge lies in optimizing of the drug-carrier interactions to achieve the ideal release kinetics to certify therapeutic effectiveness. Ensuring the safety and biocompatibility of therapeutic CLs is vital [109–111]. The CLs must be compatible with eye tissues, so that it does not cause any toxicity or unfavorable reactions, and maintain long-term eye health. Surface modifications and material selection play an important role in addressing this issue [111–114]. Achieving adequate drug bioavailability at the desired site in the eye is an important challenge [115]. Ocular barriers, such as tear film, conjunctiva, and cornea can restrict penetration and absorption of drug. Designing CLs that can increase drug permeation and overcome ocular barriers is vital for efficient drug delivery [105, 113, 116]. Therapeutic CLs must be stable during their intended wear period. They should not prematurely release or degrade drugs while being worn or during storage. Optimizing drug stability and the physical characteristics of the lenses is crucial to ensure reliable drug delivery and longevity of the lenses. CLs should offer sufficient comfort and be acceptable to users for long-term wear. They should not cause dryness, irritation, or discomfort that might influence the patient’s compliance [117]. Optimizing the lens design, surface properties, and fitting parameters to increase comfort are important challenges [118]. The transition from laboratory-scale manufacture to large-scale production of therapeutic CLs can be challenging. Ensuring reproducibility, consistent quality, and cost-effectiveness throughout mass production is crucial. Development of scalable manufacturing methods and optimization of material source are important issues. Meeting regulatory necessities and achieving essential approvals for therapeutic CLs cause some challenges. These include indicating efficacy, safety, and compliance with applicable regulations for DDS or medical devices. Conducting rigorous preclinical and clinical studies and navigating the regulatory pathway are crucial for market approval [102, 119, 120].

Achieving high sensitivity and specificity in the detection of biomarkers in tear fluid is challenging. There are ongoing efforts to identify biomarkers in tears that could enable disease diagnosis through the use of smart CLs equipped with biosensors. However, further research is required to confirm their suitability for this purpose. Additionally, clinical studies are necessary to establish a correlation between the biomarker levels in tears and the actual progression of diseases. It’s essential that these sensors exhibit a high degree of accuracy to be effectively employed in disease diagnosis. False positives or false negatives can lead to misdiagnosis [111]. Also, precise targeting of nano-based materials to specific areas of the eye affected by glaucoma can be challenging because ensuring that the materials reach the desired locations is critical for accurate diagnosis. The challenge of electrochemical detection approaches lies in methods harnessing the electric current, converting them into measurable signals, and developing the supplementary microcomponents for an electrochemical sensor [121]. Future prospects in the field of nano-based technologies will enable the development of more specific and sensitive sensors for the detection of glaucoma biomarkers, which could lead to more accurate and earlier diagnosis. Nano-based materials can also be customized to target specific biomarkers associated with an individual’s glaucoma subtype, allowing for personalized treatment plans. In addition to diagnosing glaucoma, nano-based materials are able to deliver therapeutic agents to the affected areas and thus provide a combined diagnostic-therapeutic approach. Furthermore Nano-based sensors have the ability to remotely monitor glaucoma patients, allowing doctors to track disease progression and adjust treatment plans as needed. In addition, the use of nanotechnology can improve the imaging techniques used in the diagnosis of glaucoma and provide more accurate images of eye structures [98, 122]. Addressing the above-mentioned challenges needs interdisciplinary collaborations among researchers, engineers, ophthalmologists, and regulatory experts. Continued advancements in material science, drug delivery technologies, and ocular physiology understanding are necessary to overcome these challenges and unlock the full potential of diagnostic and therapeutic CLs [49, 105, 116, 123].

## Conclusion

The use of CLs for therapeutic purposes, in addition to vision correction, has been proposed for several decades. While the adoption of this technology is still relatively limited, there is a growing body of studies demonstrating the advantages of therapeutic CLs, especially in the management of chronic eye diseases that need precise control of drug dosage, frequency, and administration mode, like glaucoma. Non-adherence to glaucoma therapy is a significant risk factor for disease progression, and therapeutic CLs offer several advantages in addressing this issue. These advantages include improved disease control, causing fewer hospitalizations and ocular surgeries, as well as reduced morbidity (blindness). Therapeutic CLs also allow for the use of smaller quantities of drugs, resulting in pharmacoeconomic benefits, while providing more patients’ convenience and therapeutic effectiveness. For patients already used CLs for optical correction, the use of CLs for glaucoma treatment can be particularly beneficial. It not only offers more convenient IOP control but also eliminates the need to remove the CLs before administering eye drops. While older populations may need some time to adjust to this system, once habituated, the use of CLs can become more comfortable and safer. Additionally, it requires less manual dexterity compared to eye drop administration. Therapeutic CLs can be designed for prolonged use, requiring only daily, weekly, or monthly changes, as supported by current studies in this field. Table 1 describes some features of the reported nanomaterial-laden CLs for diagnosis and treatment of glaucoma.

**Table 1:** Summary of the reported nanomaterial-laden contact lenses for diagnosis and treatment of glaucoma

Devise	Target	Advantages	Outcomes	Refs.
Latanoprost-eluting contact lens (CLs)	Treatment	Long-term release of latanoprost, safe in cell and animal studies	Rapid initial release of the drug followed by prolonged drug release over four weeks	[54]
Pueranin-cyclodextrin nanoparticles (NPs)-laden CL	Treatment	Significant drug loading capacity, long drug retention time	Increased retention time of pueranin-cyclodextrin NPs-laden CLs compared to 1% puerarin eye drop (77.45 min vs.12.88 min)	[55]
Timolol maleate (TM)- implant CL	Treatment	Controlled drug delivery, safe in eye irritation and cytotoxicity analysis, enhanced mean residence time compared with eye drop	Prolonged drug release for 168 h, sustained IOP reduction for 192 h in rabbit	[56].
Vitamin E-loaded pHEMA-hydrogel CLs	Treatment	Increased drug loading	Increasing the timolol and brimonidine loading on the lenses by 19.1% and 18.7%, respectively, without significant change in the duration of drug release from the lenses	[57]
latanoprost-loaded PLGA NPs-laden CLs	Treatment	Sustained delivery of latanoprost	Significantly greater IOP reduction compared to the eye drops on day 3, 5, and 8 for the high-dose CLs	[58]
Acetazolamide-loaded polymeric CLs	Treatment	Sustained drug release	Long term drug release from the nano-drug complex for 3 h with the complete destruction of the polymer matrix within 5 min	[59]
Brimonidine @LDH/Thermogel-laden CLs	Treatment	High biocompatibility, non-toxic for human corneal epithelium	In vitro sustained release for up to 144 h, in vivo sustained release for at least 7 days	[60]
Nanogel-laden bicontinuous microemulsion timolol-eluting CLs	Treatment	Good oxygen permeability and optical transmission, controlled release of drugs only when the CLs is worn	Drug release at 35 C while maintaining oxygen permeability and optical transmission	[62]
Bimatoprost/latanoprost -eluting vitamin-E modified CLs	Treatment	Good biocompatibility, extended release of drug	Extended release of bimatoprost by 10 to 40-fold. Sustained delivery of therapeutic doses for more than 10 days	[63]
Timolol maleate-loaded chitosan-alginate NPs-laden CLs	Treatment	Sustained drug delivery and improved patient compatibility	Sustained drug release for 3 days, extending up to 6 days	[64]
Bimatoprost-loaded microemulsion-laden CLs	Treatment	Low burst release, improvement in the retention time of drug	The two-fold increase in the uptake/loading of bimatoprost, improved in vitro release rate profiles up to 48 to 96 h	[66]
Timolol/latanoprost-loaded micelles-laden CLs	Treatment	Sustained release of timolol and latanoprost, improved bioavailability and residence time	Long-term release of timolol and latanoprost, respectively, for up to 120 and 96 h in tear fluid, improved mean bioavailability (2.2-fold and 7.3-fold) and residence time (79.6-fold and 122.2-fold) compared to eye drops for both timolol and latanoprost, respectively	[31]
Timolol-loaded microemulsion-laden soft CLs	Treatment	High drug loadings, controlled release of drug in deionized water	Slow drug release in deionized water for gels containing timolol-loaded microemulsions, very rapid release in PBS and in saline due to greater solubility of timolol in these solutions compared to deionized water	[68]
Travoprost-loaded microemulsion soaked CLs	Treatment	Improved drug loading, swelling and optical transmission properties	Enhanced drug loading/uptake and improved physical properties of travoprost loaded microemulsion soaked CLs compared with the traditional soaking method, slow (48–120 h) in vitro drug release, high drug retention time in the tear fluid	[69]
Timolol-loaded microemulsion-laden silicone CL	Treatment	Improved drug loading/release, and retention	2-fold improvement in timolol loading, improved drug release up to 48–96 h, improved retention time in rabbit tear fluid, prolonged IOP reduction up to 96 h	[70]
Latanoprost-loaded PEGylated solid lipid NPs- laden soft CL	Treatment	Improved swelling, safe in histopathological studies, improved drug loading and sustained drug release	High drug uptake and sustained drug release up to 96 h for LP-pSLN-L lenses, high drug concentration at all-time points up to 96 h in animal studies	[71]
Timolol maleate (TM)-loaded nanodiamond (ND)-embedded CL	Treatment	Sustained release of drug, retention of drug activity in primary human trabecular meshwork cells	Controlled and sustained release of timolol maleate in the presence of lysozyme	[76]
Timolol-loaded gold NPs (GNPs)-laden CL	Treatment	Significant loading/ uptake of drug with the GNPs without altering their physical characteristics	Significant decrease IOP (72 h), high concentration of timolol with CLs loaded with GNPs compared with the soaked CLs without GNPs, no significant enhancement in the rate of timolol release	[77]
Levobunolol-loaded eudragit NPs-laden CL	Treatment	Improved swelling properties, transmittance, sustained drug release, and enhanced drug permeation	Sustained drug release, enhanced permeation through the CLs compared to commercially available eye drops	[80]
Bimatoprost-loaded graphene oxide-laden CLs	Treatment	Improved swelling properties and transmittance of CL, controlled release of drug	Not improved bimatoprost uptake, enhanced in vitro drug release, substantial decrease in the cumulative and burst release of bimatoprost, substantial improvement in the mean residence time compared to the eye drop solution	[81]
Timolol-eluting graphene oxide-laden silicone CL	Treatment	Improved swelling properties, controlled drug release, sustained drug delivery	Significant reduction in burst release and improved release profiles compared to the respective CLs without GO, significant enhancement in mean residence time	[82]
Timolol maleate-loaded silica poly methacrylic acid (PMAA) NPs incorporated CL	Treatment	Great biocompatibility and high physical/ chemical stability, pH-responsive drug release, sustained drug release at physiological pH	No substantial release of pH-responsive timolol maleate by silica-alginate composite NPs, promising release of pH-responsive timolol maleate by silica-PMAA NPs	[83]
Brimonidine-loaded silica NPs-laden silicone CLs	Treatment	Improved oxygen permeability, transmittance, swelling, and reduced lysozyme adherence, controlled drug delivery, enhanced safety for human applications, increased convenience, and heightened adherence by patients	Controlled release of brimonidine for up to 144 h, high concentration of brimonidine for 96 h in a rabbit tear fluid model, low cumulative drug release	[84]
Drug-eluting mesoporous silica NPs-laden CLs	Treatment	Prolonged drug release, improved drug loading capacity and compatibility, safe for ocular use	Sustained release of glaucoma drugs, controlled drug release over time, good drug loading	[85]
Graphene and carbon nanotubes-laden CL	Diagnosis	Ease of fabrication, good repeatability, linearity, and accuracy in tracking fluctuating IOP levels	High sensitivity (36.01 µV mmHg − 1), capability to track dynamic pressure changes within the normal IOP range of 9 to 34 mmHg	[91]
Graphene nanowalls (GNWs)-laden CL	Diagnosis	Non-invasive technique, continuous IOP monitoring with high sensitivity and minimal power consumption	IOP monitoring with the sensitivity of 42,250 (ppm/mmHg), surpassing that of the IOP tonometer	[92]
Soft and transparent nano -based CL	Diagnosis	Real-time IOP monitoring in human eyes, excellent biocompatibility, achieving accuracy comparable to the gold-standard tonometry, stable against inflammation, thermal exposure, and electromagnetic radiation, causing minimal corneal abrasion, safe in ten human participants	Accurate quantitative measurements of IOP without inducing inflammation, providing the measurements in rabbits that match those of a commercial tonometer	[93]
Transparent silver nanowire-laden smart CLs	Diagnosis	Noninvasive and continuous IOP monitoring	Successful monitoring of IOP changes in living rabbit eyes	[95]
Gold hollow nanowire-based CLs	Diagnosis	High ocular strain sensitivity, chemical stability and biocompatibility	Successful IOP monitoring in glaucoma induced rabbits, ability for on-demand delivery of timolol to regulate IOP	[96]
Gold nanobowls (AuNBs)-based CL	Diagnosis	Convenient, noninvasive, and possibly multifunctional platform for monitoring diagnostic biomarkers in human tears	Dual-functional smart CLs sensor for IOP monitoring, quantitative analysis of MMP‐9 at a low nanomolar range in real tear samples	[97]

## Data Availability

Not applicable.
